# Bacteria in Mountain Air

**Published:** 1884-02

**Authors:** 


### BACTERIA IN MOUNTAIN AIR.

That alcoholic liquors are destructive to mind and body, that the water we drink is never free from contamination, and that much of our food is dangerously adulterated, are truths with which we have all become familiar. It was a consolation in the midst of so much impurity to reflect that in the mountains, if we could only get to them, we could at least breath pure air. But this also is vanity. Bacilli bacteria meet us at every turn; and there are microbes even in mountain air. Children after seeing at a scientific lecture the monsters contained in a drop from the filter have been known for some time afterward to hold water in abhorrence, and to refuse under all circumstances to drink it; and after reading the report of Professor Freudenreich, of Berne, and Dr. Miquel, chief of the Montsouris Observatory, on atmospheric air, the timid may almost be afraid to breathe. The air of large towns is simply unfit for human consumption; and though reluctant assent is given to Professor Tyndall's general conclusion that the air at great heights is for the most part of a high degree of purity, this does not alter the fact that "bacteria are always present in mountain air." Nevertheless there are degrees in atmospheric purity. Very good air indeed can be found at from 2,000 to 4,000 metres above the level of the sea; and the air on the surface of a Swiss lake contains bacteria only in the proportion of .85 to 500 cubic metres. The air of the land near a lake is three times more impure than that of the lake itself; the air of a room in the same locality thirty times more impure; the air of Montsouris, near Paris, 330 times more impure, and the air of the Rue Rivoli 2,400 times mcre impure. Although not pure in an absolute scientific sense, the atmosphere of the Swiss mountains is after all a little better than that of Paris.

